# Editorial: Clinic-oriented multifunctional biomaterials: from rational design to applications

**DOI:** 10.3389/fbioe.2023.1338945

**Published:** 2023-11-21

**Authors:** Meng Tian

**Affiliations:** Department of Neurosurgery and Neurosurgery Research Laboratory, West China Hospital, Sichuan University, Chengdu, China

**Keywords:** clinic, oriented, biomaterials, rational design, application

Although great success has been achieved in the development of various biomaterials, there are only a few products that can meet the requirements of clinical applications. Thus, rational design and fabrication of multifunctional biomaterials that are capable of better meeting the needs in the clinic are promising for practical application. Recently, a great quantity of biomaterials have been engineered for practical applications in view of clinical use, which could be in terms of preparation, evaluation, or clinical trials.

In this Research Topic, we collected of 11 articles, including 8 articles and 3 reviews, contributed by 82 researchers worldwide. The original research articles involved multiple biomaterials: nanolipogel, membrane, hydrogel, stent, red blood cell substitute, tracheal allograft, titanium prosthesis, and multi-layered drug delivery system. These biomaterials were rationally designed and provide enormous references for their clinical applications.

There are three review articles in this Research Topic. In their review article, Newman et al. described features of microfluidic technology with a focus on blood-contacting applications, where material hemocompatibility was discussed in the context of interactions with blood components, from the initial absorption of plasma proteins to the activation of cells and factors and the contribution of these interactions to the coagulation cascade and thrombogenesis. Finally, they reviewed the techniques for improving microfluidic channel hemocompatibility through material surface modifications, including bioactive and biopassive coatings, and future directions. In another review article, Huang et al. focused on recent progress in the use of hydrogels in mimicking the hematopoietic niche for the efficient expansion of hematopoietic stem cells, where biomimetic strategies and the combination use of hydrogel matrices and microfluidics, including the emerging organ-on-a-chip technology, are summarized. They also provided a brief description of novel stimulus-responsive hydrogels that are used to establish an intelligent dynamic cell microenvironment. Regenerating periodontal tissue is a huge clinical challenge because of the structural complexity and the poor self-healing capability of periodontal tissue, in which the homing of endogenous stem cells may bring promising treatment strategies in the future. In the third review article, Meng et al. summarized the stimulating strategies for endogenous cell homing, such as the combination of cytokines and chemokines, and the implantation of tissue-engineered scaffolds.

In this Research Topic, all of the original research presented crucial aspects regarding preparation, evaluation, and potential clinical translation applications. Zeng et al. combined a bioengineering approach and a cryopreservation technique to fabricate a neo-trachea using a pre-epithelialized cryopreserved tracheal allograft. Using rat heterotopic and orthotopic implantation models, they confirmed that tracheal cartilage has sufficient mechanical properties to bear neck movement and compression, indicating that pre-epithelialization with respiratory epithelial cells can prevent fibrosis obliteration and maintain lumen/airway patency and showing that a pedicled adipose tissue flap can be easily integrated with a tracheal construct to achieve neovascularization. Yang et al. prepared an antibacterial polypropylene/polyurethane composite membrane through a hot-pressing membrane-forming technology for invisible orthodontics application. The membranes were conferred with favorable transparency (∼70% in the visible light range) and excellent antibacterial ability. Kong et al. prepared a red blood cell substitute composed of polymerized human cord hemoglobin assisted with ascorbic acid that alleviates oxidative stress for blood transfusion.

Two articles have been collected related to improving drug delivery. Nanolipogel emerges as a potential system to encapsulate and deliver hydrophilic drugs while suppressing their initial burst release. However, there is a lack of characterization of their drug release mechanism. Melvin Liew et al. used different molecular weights of poly (ethylene glycol) diacrylate to vary the mesh size of the nanogel core, drawing inspiration from the macromolecular crowding effect in cells, which can be viewed as a mesh network of undefined sizes. The multi-layered drug delivery system has promising potential to achieve controlled release. However, existing technologies face difficulties regulating the number of layers and layer–thickness ratio. Zheng et al. utilized layer-multiplying co-extrusion technology to modulate the layer–thickness ratio and drug release to expand their application.

Another two articles have been published to focus on *in vivo* implantation evaluation. The ultrathin-strut drug-eluting stent (DES) has shown better clinical results than thin- or thick-strut DES. Hahn et al. investigated whether re-endothelialization was different among three types of commercial DES, and their results showed that re-endothelialization after stent implantation was related to smooth muscle cells (SMC) coverage and SMC layer differentiation, in which ultrathin-strut DES exhibited significantly faster and denser re-endothelialization. Currently, there is no ideal material available for posterior scleral reinforcement (PSR) to prevent the progression of high myopia. Xu et al. evaluated the safety and biological reactions of robust regenerated silk fibroin hydrogels as potential grafts for PSR in animal experiments.

Last, we highlight one article collected that reported a clinical trial. Total wrist arthroplasty is an effective treatment for end-stage wrist arthritis from all causes. However, wrist prostheses are still prone to complications such as prosthesis loosening and periprosthetic fractures after total wrist arthroplasty. Cai et al. designed and developed a personalized three-dimensional printed microporous titanium artificial wrist prosthesis (3DMT-Wrist) for the treatment of end-stage wrist joint and investigated its safety and effectiveness.

In conclusion, the current Research Topic reports different types of biomaterials and their broad applications, providing new chances for meeting requirements toward clinical translation.

